# Aerobic and Anaerobic Biodegradability of Organophosphates in Activated Sludge Derived From Kitchen Garbage Biomass and Agricultural Residues

**DOI:** 10.3389/fbioe.2021.649049

**Published:** 2021-02-17

**Authors:** Xingfeng Yang, Deling Fan, Wen Gu, Jining Liu, Lili Shi, Zhi Zhang, Linjun Zhou, Guixiang Ji

**Affiliations:** ^1^College of Modern Agriculture and Ecological Environment, Heilongjiang University, Harbin, China; ^2^Nanjing Institute of Environmental Science, Ministry of Ecology and Environment, Nanjing, China

**Keywords:** organophosphates, biomass, biodegradation, aerobic, anaerobic, active sludge, kitchen garbage

## Abstract

Organophosphates (also known as organophosphate esters, OPEs) have in recent years been found to be significant pollutants in both aerobic and anaerobic activated sludge. Food waste, such as kitchen garbage and agricultural residues, can be used as co-substrates to treat the active sludge in sewage treatment plants (STPs). We investigated the biodegradability of nine OPEs derived from kitchen garbage biomass and agricultural residues under different conditions. Under anaerobic conditions, the rate of removal of triphenyl ester OPEs was significantly higher than that of chloride and alkyl OPEs. The addition of FeCl_3_ and Fe powder increased the rate of degradation of triphenyl ester OPEs, with a DT_50_ for triphenyl ester OPEs of 1.7–3.8 d for FeCl_3_ and 1.3–4.7 d for Fe powder, compared to a DT_50_ of 4.3–6.9 d for the blank control. Addition of an electron donor and a rhamnolipid increased the rate of removal of chlorinated OPEs, with DT_50_ values for tris(2-carboxyethyl)phosphine) (TCEP) and tris(1,3-dichloroisopropyl)phosphate (TDCPP) of 18.4 and 10.0 d, respectively, following addition of the electron donor, and 13.7 and 3.0 d, respectively, following addition of the rhamnolipid. However, addition of an electron donor, electron acceptor, surfactant, and Fe powder did not always increase the degradation of different kinds of OPEs, which was closely related to the structure of the OPEs. No treatment increased the removal of alkyl OPEs due to their low anaerobic degradability. Tween 80, a non-ionic surfactant, inhibited anaerobic degradation to some degree for all OPEs. Under aerobic conditions, alkyl OPEs were more easily degraded, chlorinated OPEs needed a long adaptation period to degrade and finally attain a 90% removal rate, while the rates of degradation of triphenyl ester OPEs were significantly affected by the concentration of sludge. Higher sludge concentrations help microorganisms to adapt and remove OPEs. This study provides new insights into methods for eliminating emerging pollutants using activated sludge cultured with kitchen garbage biomass and agricultural residues.

## Introduction

Sewage treatment plants (STPs) are the major secondary sources of emerging pollutants, which may not be completely removed or degraded (Styszko et al., 2020). Many pollutants pass through STPs owing to their persistence or continuous release or to the inefficient operation of STPs (Saxena et al., 2021).

The aerobic and anaerobic biodegradation of these pollutants are the major removal mechanisms employed in STPs. However, many full-scale STPs operate with low efficiencies, due to an unbalanced nutrient ratio, deficiencies in essential elements, an accumulation of volatile fatty acids, and the presence of process inhibitors (Tonanzi et al., 2020).

To overcome these problems, food waste, such as kitchen garbage with a high C/N ratio, is generally used as a co-substrate with municipal waste activated sludge. This method can overcome the difficulties associated with treating nutrient-deficient activated sludge, to adjust its unbalanced *C*/*N* ratio, and to increase buffer capacity, dilute toxic compounds, and adjust micro- and macro-nutrient availability (Z.-l. Zhang et al., 2013).

Organophosphoric acid esters (OPEs), one of the most commonly used organophosphorus flame retardants (Eede et al., 2012), have been used as plasticizers and flame retardants in plastics, electronic equipment, furniture, textiles, construction, and transport (Martínez-Carballo et al., 2007). Table 1 lists the various types of OPEs and specific information related to them. OPEs have been confirmed to possess both acute and chronic toxicities, including eye and skin irritation, neurotoxicity, reproductive toxicity, endocrine disruptive effects, carcinogenicity (Castro-Jimenez et al., 2014), and environmental biological toxicity and risk (Kai, 2005). OPEs from urban, industrial, agricultural, street-flushed sewage, and atmospheric dry and wet depositions (Tappe et al., 2002; Ratola et al., 2012; O’Brien et al., 2015; Saini et al., 2016) all eventually end up in STPs.

**TABLE 1:** Physicochemical properties of nine types of OPEs.

Compound	Abbreviation	CAS number	Chemical formula	Molecular weight	lg *K*_*oc*_	lg *K*_*ow*_
Tripropyl phosphate	TPrP	513-08-6	C_9_H_21_O_4_P	224.23	2.58	1.87
Tri-isobutyl phosphate	TiBP	126-71-6	C_12_H_27_O_4_P	266.31	3.14	3.60
Tributyl phosphate	TBP	126-73-8	C_12_H_27_O_4_P	266.31	3.37	4.00
Tris(2-chloroethyl) phosphate	TCEP	115-96-8	C_6_H_12_Cl_3_O_4_P	285.49	2.58	1.44
Tris(1,3-dichloro-2-propyl) phosphate	TDCP	13674-87-8	C_9_H_15_Cl_6_O_4_P	430.9	4.04	3.65
Triphenyl phosphate	TPhP	115-86-6	C_18_H_15_O_4_P	326.28	3.47	4.59
Tri-*o*-cresyl phosphate	*o*-TTP	78-30-8	C_21_H_21_O_4_P	368.36	4.67	5.11
Tri-*m*tolyl phosphate	*m*-TTP	563-04-2	C_21_H_21_O_4_P	368.36	4.64	6.43
Tri-*p*-tolyl phosphate	*t*-TTP	78-32-0	C_21_H_21_O_4_P	368.36	4.20	6.34

Kim et al. (2017) compared the entry of OPEs into U.S. STPs with reported production and showed that the mass loads of triphenyl phosphate (TPHP), tris(1-chloro-2-propyl) phosphate (TCIPP), tris(1,3-dichloro-2-propyl) phosphate (TDCIPP), and tri(*n*-butyl) phosphate (TNBP) in STPs were 1.3–2.8% of the annual output. Anneli et al. (2005) showed that 15% of domestic OPE usage in Sweden is discharged into STPs. Therefore, the study of OPE biodegradation in both aerobic and anaerobic processes in STPs is vitally important.

There are few studies that have examined the best conditions of oxygen availability for OPE degradation, and most have focused mainly on the investigation of the rate of removal of OPEs from STPs. Only a few studies have pointed out that the degradation of non-chlorine OPEs occurs mainly in aerobic aeration tanks. In addition, there was no obvious removal of alkyl OPEs under anaerobic conditions (Kawagoshi and Fukunaga, 1994). The study of suitable oxygen conditions for the degradation of different OPEs has practical significance for guiding the clean-up and maintenance of STPs.

The addition of electron donors and electron acceptors is also important for the biodegradation of organophosphate pollutants (Kieft et al., 1999; Gou et al., 2019). The biodegradation of chemicals depends largely on the availability of electron receptors and their respective energy yields. The removal of chemical substances can be improved by electron receptor species such as O_2_, Fe^3+^, CO_2_, CO, NO_3_, NO_2_, NO, N_2_O, SO_4_^2–^, and S (Häggblom and Young, 1999; Amend and Shock, 2001; Wu et al., 2017). In aerobic environments, microorganisms generally use oxygen as an electron receptor to accelerate the biodegradation process. In anaerobic degradation, however, because of the lack of oxygen as electron acceptor, microorganisms must use alternative electron receptors such as sulfate, nitrate, and trivalent iron, which are usually supplied more economically than oxygen (Varjani and Upasani, 2017).

In addition to the need for electron acceptors, methanogens rely on electron donors and matrixes to degrade these complex organic chemicals, such as H_2_, Fe^2+^, H_2_S, sulfide minerals, CH_4_, various mono- and dihydroxyl carboxylic acids, alcohols, amino acids, and complex organic substrates (Grishchenkov et al., 2000). Electron donors, also known as co-matrixes, are added primarily as nutrients for anaerobic treatment, contaminants are generally removed by co-metabolism, sufficient nutrient substrates are made available, and anaerobic sludge can produce sufficient enzymes to degrade organic pollutants (Wei et al., 2010; Cao et al., 2012).

Zero-valent iron and surfactants can also be added to the anaerobic environment to promote anaerobic biodegradation. Because of its high reduction activity, zero-valent iron is often used to treat all kinds of pollutants in water. Zero-valent iron can provide electrons for microorganisms and keep the redox potential of the anaerobic environment low (Völker et al., 2017). Studies have shown that the presence of iron chips (1 g⋅L^–1^) in water can significantly increase the anaerobic degradation of Chlorpyrifos and Bisphenol A (Shi et al., 2019b; Yang et al., 2019). Iron is also a major protein cofactor that is essential for most organisms and increases the production of degrading enzymes (Beauchene et al., 2015). Because of its advantages in accelerated hydrolysis, fermentation, and anaerobic digestion, zero-valent iron increases the abundance of methanogens and promotes methane production (Wei et al., 2018; Pan et al., 2019).

Surfactants can also increase the rate of degradation of organic pollutants, which can overcome the diffusion limitation of substrates to cells, reduce the tension and viscosity of the water interface effectively, and increase bioavailability. There are many kinds of surfactants, including biological, anionic, and non-ionic surfactants. Biosurfactants are produced mainly by microorganisms, which contribute to the desorption of soil pollutants and their migration to microbial cells, resulting in the reconstruction of their surfaces and a change in bioavailability of biodegradable compounds (Zdarta et al., 2018). The non-ionic surfactant Tween 80 and the rhamnolipid biosurfactant both enhance the enzyme activities of amylase, carboxymethyl cellulase, and xylanase effectively (Zeng et al., 2006), improving enzyme stability and increasing the enzymatic reaction rate (Mo et al., 2008).

Most studies in this area have examined the removal of emerging pollutants in unconditioned active sludge from STPs. However, there is a lack of information regarding the role of active sludge cultured with kitchen garbage and agricultural residues. In this study, the biodegradability of nine OPEs in both aerobic and anaerobic activated sludge derived from kitchen garbage biomass and agricultural residues was investigated under different conditions, including oxygen availability and addition of electron donors, electron acceptors, or surfactants, etc. The results were compared in order to determine the optimal degradation conditions, which will provide a new perspective on the effect of activated sludge cultured with kitchen garbage and agricultural residues to enable the elimination of emerging pollutants.

## Materials and Methods

### Chemicals

The OPE standard substances, including trimethyl phosphate (TMP), tripropyl phosphate (TPrP), tri-isobutyl phosphate (TiBP), tributyl phosphate (TBP), Tris(2-chloroethyl) phosphate (TCEP), tris(1,3 dichloropropyl) phosphate (TDCPP), triphenyl phosphate (TPhP), *o*-trimethylphenol phosphate (*o*-TT), tri-*m*-cresyl phosphate (*m*-TTP), tri-*p*-cresyl phosphate (*p*-TTP), were purchased from Balinway Chemical Reagent Co., Ltd. (China). The physicochemical properties of the nine kinds of OPEs are listed in Table 1.

HPLC-grade *n*-hexane (Hex), ethyl acetate (EtAc), and acetone (ACE) were provided by Merck & Co. (Darmstadt, Germany).

NaSO_4_ (AR), NaCl (GR), KH_2_PO_3_ (GR), Na_2_HPO_4_⋅12H_2_O (AR), NH_4_Cl (GR), NaNO_3_ (AR), CaCl_2_⋅2H_2_O (GR), MgCl_2_⋅6H_2_O (AR), Fe powder, Tween 80, FeCl_3_ (GR), NaHCO_3_ (GR), rhamnolipid, FeCl_2_⋅4H_2_O (AR), Na_2_S⋅9H_2_O (GR), HgSO_4_ (GR), and resazurin (C_12_H_7_NO_4_) were purchased from China National Pharmaceutical Group Corporation (Sinopharm, Beijing, China).

### Sample Collection

Both aerobic and anaerobic activated sludges were collected from aerobic and anaerobic ponds of the Nanjing Chengdong STP. The treatment process was A^2^/O and the volume of sewage was 350,000 m^3^, serving approximately 500,000 people.

The kitchen garbage and agricultural residues obtained from school canteens were composed of rice, meat, and small quantities of vegetables. The garbage was chopped and then diluted to 82.75 g L^–1^ with tap water. A substrate blend of waste activated sludge and kitchen garbage was prepare in a ratio of 5:1 by volume. The blended sludges was cultivated under aerobic and anaerobic conditions at room temperature.

### Biodegradation Experiments

The medium was prepared with deionized water, to which was added 0.27 g⋅L^–1^ anhydrous potassium dihydrogen phosphate (KH_2_PO_4_), 1.12 g⋅L^–1^ disodium hydrogen phosphate dodecahydrate (Na_2_HPO_4_⋅12H_2_O), 0.53 g⋅L^–1^ ammonium chloride (NH_4_Cl), 0.075 g⋅L^–1^ calcium chloride dihydrate (CaCl_2_⋅2H_2_O), and 0.10 g⋅L^–1^ magnesium chloride hexahydrate (MgCl_2_⋅6H_2_O).

The aerobic and anaerobic sludge cultures were dispersed in the medium so as to prepare sludge suspensions whose pH was adjusted to 7 with NaOH.

#### OPE Volatilization Experiments

A 3 L wash bottle was loaded with 1.5 L of deionized water. Nine types of OPEs listed in Table 1 were added to a wash bottle to a final concentration of 1 mg⋅L^–1^. The air inlet of the wash bottle was connected to an aeration device set at a flow rate of 500 ml⋅min^–1^. The outlet of the aeration device was connected to a wash bottle containing methanol to absorb the volatile OPEs.

#### Aerobic Biodegradation

An OPE stock solution was added to an Erlenmeyer flask to a concentration of 0.5 mg⋅L^–1^ and the flask was placed in a fume hood to volatilize the acetone to dryness. The sludge suspension was added so that the sludge concentration was 1 g⋅L^–1^ for the low sludge concentration treatment and 10 g⋅L^–1^ for the high sludge concentration treatment, with 10 parallel treatments for each concentration. After the addition was complete, the rubber plug was covered and kept aerated and placed on a shaker for culture at 30°C. Samples were collected and analyzed intervals.

#### Anaerobic Biodegradation

The OPE stock solution was added to the prepared anaerobic fermentation bottle, the concentration was adjusted to 0.5 mg⋅L^–1^, and the bottle was then placed under the ventilation cabinet until the acetone was volatilized to dryness. The configured medium was subjected to eight different treatments, designated b, c, d, e, f, g, h, and i. Then, the sludge and additional agents were added to each treatment, as given in Table 2 (reducing iron powder, which is insoluble in water, was added separately to 12 vials). Each treatment had 12 bottles and a final volume of 100 mL. Following filling of each anaerobic fermentation bottle, nitrogen was blown through each one for 5 min to remove all traces of oxygen. Following this treatment, 0.04 g⋅L^–1^ ferrous chloride (FeCl_2_⋅4H_2_O) and 0.20 g⋅L^–1^ nine-water sodium sulfide (Na_2_S⋅9H_2_O) were added to each vial, and then 0.002 g⋅L^–1^ resazurin was added as an oxygen indicator. Under abiotic control, the medium was heated to 100°C. Then, 1 g⋅L^–1^ mercury sulfate was added after cooling to kill the microorganisms in the medium. All of the anaerobic fermentation bottles were then placed in an incubator at 35°C and shaken 1–2 times a day to release the gas produced by anaerobic fermentation. Samples were taken at regular intervals for analysis.

**TABLE 2:** Test treatments.

Group	Treatment	Symbol	Sludge (g⋅L^–1^)	Addition of substancess
Anaerobic	Biological control	b	3	–
	Electron donor	c	3	Sodium acetate: sodium propionate: sodium butyrate: sodium lactate = 1:1:1:1 (200 mmol⋅L**^–^**^1^)
	Electron receptor	d	3	NaNO_3_ = 20 mM
		e	3	Na_2_SO_4_ = 20 mM, NaHCO_3_ = 20 mM
		f	3	FeCl_3_ = 20 mM, NaHCO_3_ = 20 mM
	Surfactants	g	3	Tween 80, CMC = 0.010 mM
		h	3	Ribose glycolipid, CMC = 0.5 mM
	Reducing iron	i	3	Reducing iron powder = 2 g⋅L^–1^
Aerobic	Low sludge concentration	1 g⋅L^–1^	0.1	–
	High sludge concentration	10 g⋅L**^–^**^1^	1	–
Abiotic control	–	AC	–	Mercury sulfate = 1 g⋅L**^–^**^1^

### Analytical Methods

Samples of water (10 mL) were taken in the volatility experiments, to which were added 0.5 g of sodium chloride and 10 mL of organic solvents (*n*-hexane and ethyl acetate 1:1). The samples were then shaken for 3 min. The cap on the bottle was opened and the water and organic phases allowed to settle out. The upper organic phase was then sampled (10 mL) with a pipette, dehydrated with anhydrous sodium sulfate, and finally filtered through filter paper. Following filtering through a 0.22 μm microporous filter membrane, the samples were analyzed on a TSQ^TM^ 9000 Triple Quadropole GC-MS/MS System (Thermo Fisher Scientific, Waltham, MA, United States). The methanol receiving tail gas was also analyzed by similar means.

The samples of aerobic and anaerobic biodegradation were poured into a separate funnel, to which 3 g of sodium chloride was added, and extracted twice with 20 mL of solvent (*n*-hexane and ethyl acetate 1:1). Following dehydration with anhydrous sodium sulfate and filtration through filter paper, the organic phase was adjusted to 50 mL with *n*-hexane. The sample was then analyzed by GC-MS/MS.

The chromatographic column was a HP-5MS (30 m × 0.25 mm × 0.25 μm). The heating procedure was as follows: the starting temperature was set at 50°C and maintained for 2 min, then heated to 150°C at a rate of 20°C min^–1^ and maintained there for 4 min, then heated to 250°C at 25°C⋅min^–1^, maintained there for 1 min, then heated to 280°C at rate of 5°C⋅min^–1^ and maintained there for 3 min. Electron collision ionization (EI) sources and selected reaction monitoring (SRM) were employed. The carrier gas was high-purity helium gas, using pulse no shunt injection. The linear correlation between the standard curves of the nine OPEs was good, with an *R*^2^ value above 0.993. The detection limits of the OPEs were in the range 0.01–1.5 μg⋅L^–1^, and the quantitative limits were in the range 0.01–4.6 μg⋅L^–1^.

### Statistical Analyses

The data were collected and analyzed using Microsoft Excel 2010 (Microsoft Corporation, Redmond, WA, United States).

## Results

In the abiotic treatment, the OPE concentration remained basically unchanged, and degradation basically did not occur.

### OPE Volatility Experiments

**FIGURE 1:**
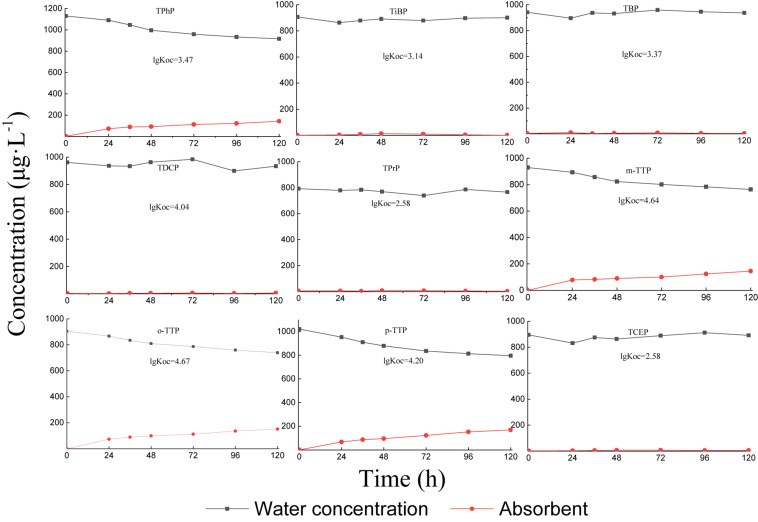
OPE concentrations in water and absorbent.

The volatility test results are shown in Figure 1. Under aeration conditions, the different types of OPEs in water showed different volatilities. The triphenyl ester OPEs (TPhP, *m*-TTP, *o*-TTP, and *p*-TTP) had a certain volatility, reaching 10–20% volatilization at 120 h. Alkyl and chlorinated OPEs were almost non-volatile.

### Anaerobic Treatment

Resazurin was added as an oxygen indicator in the anaerobic treatment. During the test, none of the anaerobic fermentation bottles showed any color, so they met the conditions of complete anaerobic fermentation. When gas was released each day, a large volume of methane was released, which also indicated that the microorganisms were metabolically active and growing normally.

#### Degradation of Triphenyl Ester OPEs

**FIGURE 2:**
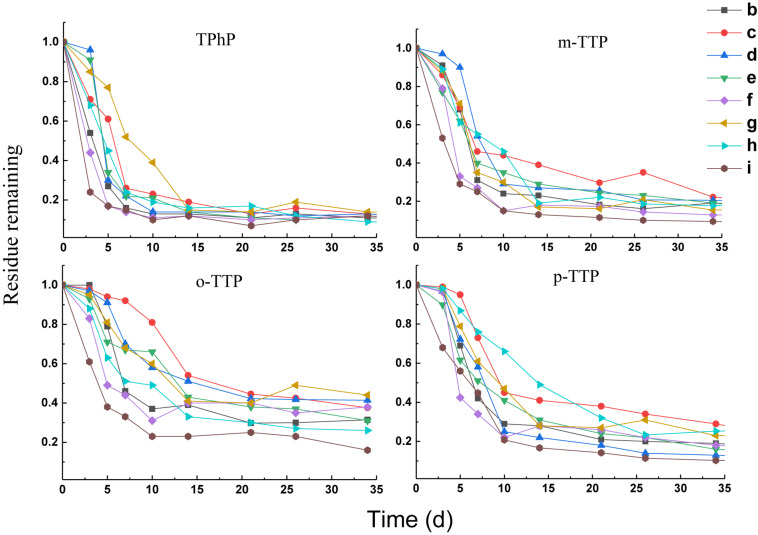
Degradation curves of triphenyl ester OPEs.

Four triphenyl ester OPEs showed similar patterns of removal in the different treatments (Figure 2). Removal occurred mainly within 21 days of the start of the experiment. The rate of removal tended to be stable after 21 days. The triphenyl ester OPEs had high removal rates in the different treatments at 21 days; that for TPhP was 83–93%, that for *m*-TTP was 70–88%, that for *o*-TTP was 55–75%, and that for *p*-TTP was 68–86%. When FeCl_3_ (treatment f) and Fe (treatment i) were added, the degradation rate was increased compared with other treatments, and the final removal rate was also significantly higher than for the other treatments.

Kinetic curves for first-order degradation were used to fit the removal rates of the OPE (Table 3). The *R*^2^ value was in the range 0.74–0.98. The DT_50_ values of the triphenyl ester OPEs with added FeCl_3_ (treatment f) were in the range 1.7–3.8 days, and with added Fe powder (treatment i) were 1.3–4.7 days, compare to a DT_50_ of 4.3–6.9 days for the blank control (treatment b). The addition of either FeCl_3_ or Fe powder increased the rate of degradation significantly. However, the other treatments did not have a significant effect on the degradation of the triphenyl ester OPEs.

**TABLE 3:** Degradation rates and DT_50_ values of triphenyl ester OPEs.

Treatment	TPhP			*m*-TTP			*o*-TTP			*p*-TTP			Promoting effect
	*k* (d^–1^)	*R*^2^	DT_50_ (d)	*k* (d^–1^)	*R*^2^	DT_50_ (d)	*k* (d^–1^)	*R*^2^	DT_50_ (d)	*k* (d(-1)	R2	DT50 (d)	
b	0.16	0.74	4.3	0.11	0.77	6.0	0.15	0.87	4.8	0.10	0.78	6.9	/
c	0.21	0.89	3.4	0.13	0.92	5.3	0.05	0.94	14.2	0.11	0.77	6.3	±
d	0.20	0.84	3.4	0.11	0.89	6.2	0.10	0.94	6.9	0.11	0.93	6.1	±
e	0.24	0.80	2.8	0.16	0.96	4.3	0.09	0.97	7.9	0.13	0.93	5.5	±
f	0.40	0.98	1.7	0.22	0.92	3.1	0.26	0.86	2.6	0.18	0.86	3.8	+
g	0.15	0.81	4.6	0.18	0.83	3.9	0.13	0.91	5.4	0.13	0.79	5.2	±
h	0.20	0.97	3.4	0.14	0.93	5.1	0.14	0.97	5.0	0.08	0.90	9.2	±
i	0.55	0.96	1.3	0.24	0.98	2.9	0.25	0.98	2.7	0.15	0.98	4.7	+

#### Degradation of Chlorinated OPEs

**FIGURE 3:**
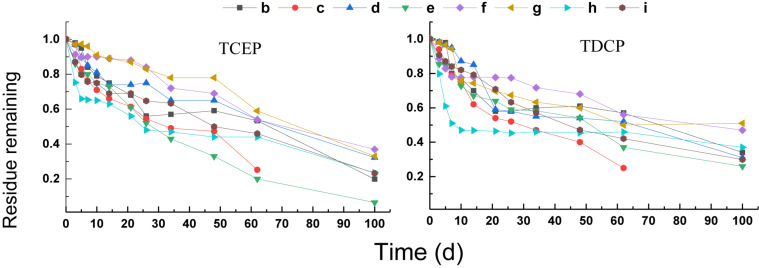
Degradation curves of chlorinated OPEs.

Compared with the triphenyl ester OPEs, the degradation of chlorinated OPEs in the different treatments was slower under anaerobic conditions, and the removal of chlorinated OPEs failed to reach 80% even after 60 days. The results of these treatments are shown in Figure 3. Different treatments had significant positive or negative effects on the degradation of chlorinated OPEs. Among them, the final rate of degradation for TCEP and TDCP in rhamnolipid (treatment h) was increased by about 10% compared to the control (treatment b). The removal rate of TCEP and TDCP was also significantly increased in treatment c of the electron donor (sodium acetate + sodium propionate + sodium butyrate + sodium lactate). At 60 days, the removal rate of TCEP was increased by 28%, and the removal rate of TDCP was increased by 31%. The effect of adding Na_2_SO_4_ (treatment e) on the removal of the two chlorinated OPEs was different: the removal rate of TCEP was increased by approximately 30%, but the removal of TDCP was inhibited.

First-order kinetic curves were used to fit the rates of degradation of chlorinated OPEs, and the results are given in Table 4. These results show that *R*^2^ was in the range 0.84–0.99. In the blank control (treatment b), the DT_50_ values of TCEP and TDCP were 38.2 and 12.3 days, respectively. In treatment c, the DT_50_ value of TCEP and TDCP were reduced to 18.4 and 10.0 days, respectively. In treatment h, the DT_50_ values of TCEP and TDCP decreased to 13.7 and 3.0 days, respectively. The results showed that the addition of an electron donor (treatment c) and rhamnolipid (treatment h) significantly increased the rate of degradation of chlorinated OPEs.

**TABLE 4:** Degradation rates and DT_50_ values of chlorinated OPEs.

Treatment	TCEP			TDCP			Promoting effect
	*k* (d^–1^)	*R*^2^	DT_50_ (d)	*k* (d^–1^)	*R*^2^	DT_50_ (d)	
b	0.02	0.89	38.2	0.06	0.85	12.3	/
c	0.04	0.99	18.4	0.07	0.92	10.0	+
d	0.01	0.93	66.8	0.03	0.92	21.4	−
e	0.02	0.99	33.9	0.02	0.94	30.3	±
f	0.02	0.96	43.7	0.01	0.86	65.4	−
g	0.01	0.97	49.0	0.05	0.94	14.9	−
h	0.05	0.84	13.7	0.23	0.95	3.0	+
i	0.01	0.93	59.1	0.02	0.99	31.6	−

The addition of Na_2_SO_4_ (treatment e) had different effects on the two chlorinated OPEs. The DT_50_ of TCEP was reduced to 33.9 days, while that of TDCP was increased to 33.3 days. Other treatments, including NaNO_3_ as electron receptor (treatment d) and FeCl_3_ as electron receptor (treatment f), non-ionic surfactants (treatment g), and reduced Fe powders (treatment i), all inhibited the degradation of chlorinated OPEs, which was very pronounced.

#### Degradation of Alkyl OPEs

**FIGURE 4:**
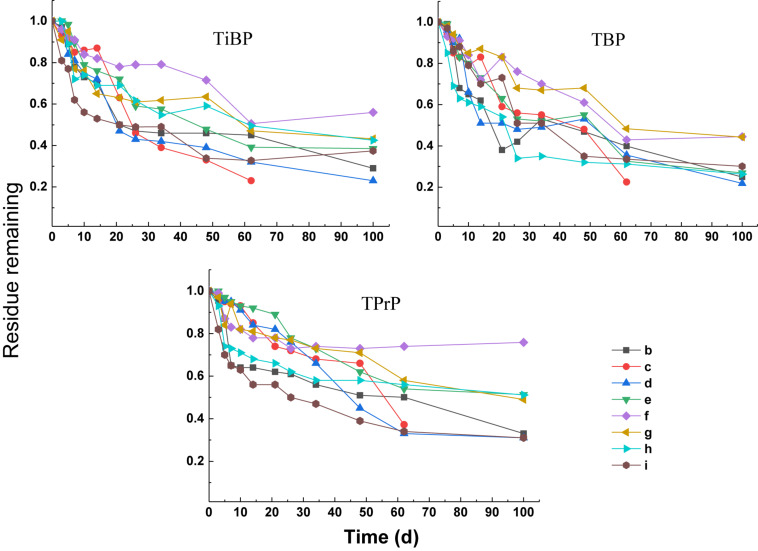
Degradation curves of alkyl OPEs.

The degradation curves of the three alkyl OPEs subjected to different treatments are shown in Figure 4. At the end of the test (100 days), the removal percentage of the three alkyl OPEs were below 78%. The removal percentage in the blank control (treatment b) was 67–75%, while the removal percentage in the electron donor (treatment c) and electron acceptor (treatments d, e, and f) were 24–74%. Therefore, all of the treatments with addition of electron donor and electron acceptor inhibited the removal of alkyl OPEs.

First-order kinetic curves were used to fit the degradation rates of the alkyl OPEs, and the results are given in Table 5. The values of *R*^2^ were in the range 0.79–0.97. In the blank control (treatment b), the DT_50_ of TiBP, TBP, and TPrP were 11.8, 8.4, and 9.7 days, respectively. The removal of alkyl OPEs under the different treatments was more complicated, and all of the treatments failed to significantly improve the degree of removal. Treatment c (sodium acetate + sodium propionate + sodium butyrate + sodium lactate), treatment d (NaNO_3_), and treatment e (Na_2_SO_4_) had an inhibitory effect on the removal of alkyl OPEs. The addition of FeCl_3_ (treatment f) had an inhibitory effect on the removal of TiBP and TBP, but no inhibition of TPrP was observed. Tween 80 (treatment g) significantly inhibited the removal of TBP and TPrP, and the DT_50_ increased to 36.7 and 43.7 days, respectively. However, the addition of the biological surfactant rhamnolipid (treatment h) had no significant effect on OPE removal. The addition of Fe powder (treatment i) inhibited the removal of TBP, increasing the DT_50_ from 8.4 to 20.4 days, while Fe powder increased the removal of TiBP and TPrP slightly, reducing the DT_50_ of TiBP from 11.8 to 6.8 days and that of TPrP from 9.7 to 5.8 days.

**TABLE 5:** Degradation rates and DT_50_ values of alkyl OPEs.

Treatment	TiBP			TBP			TPrP			Promoting effect
	*k* (d^–1^)	*R*^2^	DT_50_ (d)	*k* (d^–1^)	*R*^2^	DT_50_ (d)	*k* (d^–1^)	*R*^2^	DT_50_ (d)	
b	0.06	0.95	11.8	0.08	0.87	8.4	0.07	0.83	9.7	/
c	0.02	0.94	32.1	0.02	0.93	46.0	0.02	0.93	38.4	−
d	0.05	0.97	14.7	0.06	0.86	11.8	0.03	0.87	26.2	−
e	0.04	0.97	19.7	0.03	0.95	21.5	0.02	0.96	39.4	−
f	0.02	0.88	34.1	0.02	0.91	37.8	0.09	0.79	7.8	−
g	0.06	0.88	11.7	0.02	0.94	36.7	0.02	0.91	43.7	−
h	0.06	0.89	11.9	0.07	0.94	9.7	0.11	0.91	6.4	±
i	0.10	0.93	6.8	0.03	0.96	20.4	0.12	0.88	5.8	±

### Aerobic Treatments

During the aerobic treatment, aeration was vigorous and the dissolved oxygen concentration was greater than 3 mg⋅L^–1^, which met the requirements of aerobic degradation.

#### Aerobic Treatment of Triphenyl Ester OPEs

**FIGURE 5:**
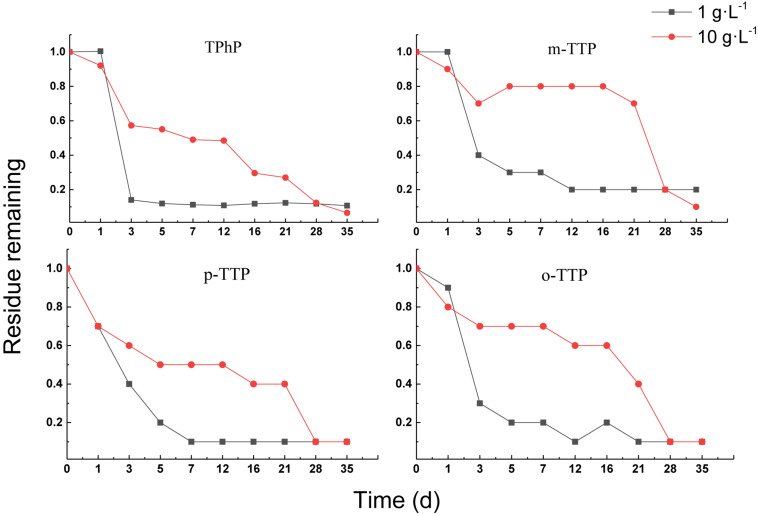
Aerobic degradation of triphenyl ester OPEs with different sludge concentration.

Figure 5 shows that the triphenyl ester OPEs were removed satisfactorily under aerobic conditions. The removal percentages of four OPEs were greater than 80% for both treatments at 35 days. The removal rate of OPEs in a sludge concentration of 1 g⋅L^–1^ was always higher than in a sludge concentration of 10 g⋅L^–1^ up to 28 days. The removal of OPEs in a sludge concentration of 1 g⋅L^–1^ was significantly greater than in a concentration of 10 g⋅L^–1^, which may have been attributed to volatilization of the triphenyl ester OPEs. The higher sludge concentration resulted in more triphenyl ester OPEs being absorbed, and the removal by volatilization in the water phase was slowed down. With increasing hydrophobicity (sorption) of the OPE, the fraction of freely dissolved OPE present in the water phase available for degradation decreases, and therefore the overall rate constant should also decrease (Hansen et al., 2017).

In contrast, the removal percentage increased rapidly in the sludge concentration of 10 g⋅L^–1^ after 28 days, and the removal percentage was higher than that in the sludge concentration of 1 g⋅L^–1^. This may have been due to the fact that, after a period of adaptation, a high sludge concentration adapts to triphenyl ester OPEs faster than a low sludge concentration.

#### Aerobic Treatment of Chlorinated OPEs

**FIGURE 6:**
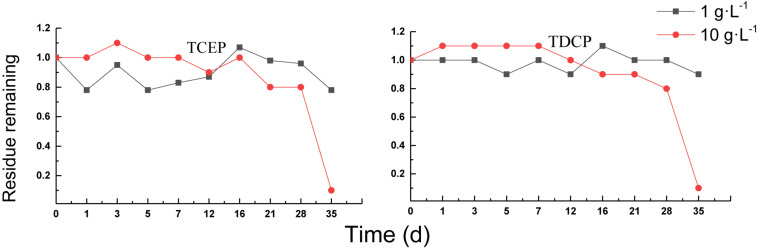
Aerobic degradation of chlorinated OPEs with different sludge concentration.

The degradation curves of chlorinated OPEs in aerobic sludge are shown in Figure 6. The removal percentages of TCEP and TDCP were different from those of triphenyl esters OPEs. In the treatment of a sludge concentration of 1 g⋅L^–1^, the removal percentages of the two chlorinated OPEs were low, with almost no removal occurring. In the sludge with 10 g⋅L^–1^, there was almost no removal after 16 days. Thereafter, the residual concentration began to decrease, and the removal percentage reached 90% by 35 days, indicating that sludge acclimation had been completed and chlorinated OPEs had begun to biodegrade.

#### Aerobic Treatment of Alkyl OPEs

**FIGURE 7:**
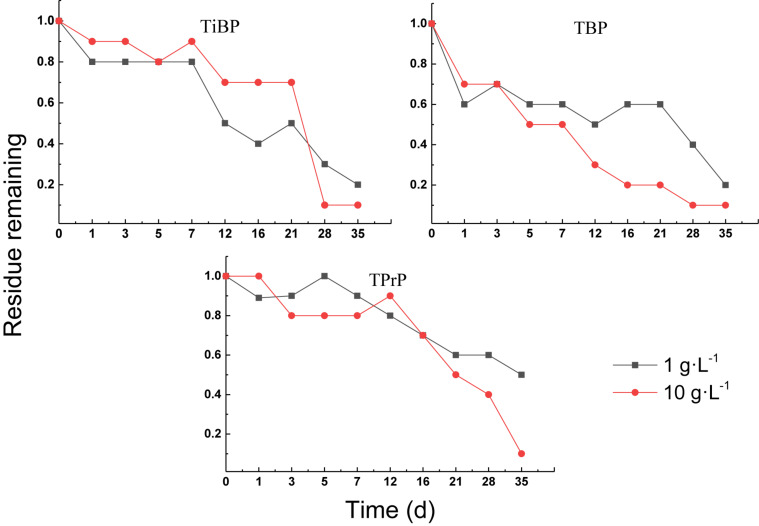
Aerobic degradation of alkyl OPEs with different sludge concentration.

The degradation curves of the alkyl OPEs in aerobic sludge are shown in Figure 7. All of the alkyl OPEs showed similar removal behavior, and the aerobic sludge was able to remove alkyl OPEs efficiently. The degree of removal of the three alkyl OPEs reached 90% in the 10 g⋅L^–1^ sludge concentration after 35 days. The removal percentages in the sludge concentration of 1 g⋅L^–1^ were slightly lower, namely, 84% for TiBP, 76% for TBP, and 51% for TPrP.

## Discussion

### Comparison of the Results Under Aerobic and Anaerobic Conditions

Under anaerobic conditions, the removal percentage of triphenyl ester OPEs was significantly higher than that of chlorinated and alkyl OPEs, indicating that triphenyl ester OPEs were more prone to anaerobic biodegradation. Facultative or specific anaerobes can couple various electron receptors to mineralize aromatic compounds through anaerobic respiration and fermentation, with higher degrees of removal (Carmona et al., 2009; Grishchenkov et al., 2000). Margot et al. (2015)’s studies have also pointed out that triphenyl ester OPEs can be successfully removed (>70%) by anaerobic degradation in STPs. The removal percentage of chlorinated OPEs was low, which may have been due to the strong electron absorption effect of chlorine atoms, which reduced the electrophilic properties of relevant enzymes (Pang et al., 2018). Studies have also shown that TCPP and TCEP in chlorinated OPEs are non-degradable and persistent in underground aquifers (Regnery et al., 2011). Kawagoshi and Fukunaga (1994) have also pointed out that there was no significant removal of alkyl OPEs under anaerobic conditions.

The removal percentages of the three types of OPEs in aerobic sludge were significantly higher than those in anaerobic sludge, which may have been due to the easier removal of OPEs under aerobic reactions. Fries and Puttmann (2003) pointed out that aerobic removal of OPEs is greater than that of anaerobic removal. The most rapid and complete degradation of most pollutants is achieved under aerobic conditions (Mezzanotte et al., 2003; Fritsche and Hofrichter, 2005). The rate of degradation of chemicals under aerobic and anaerobic conditions is related mainly to enzymes and their reactions. For example, under aerobic conditions, oxygenase activity is higher, and oxidation reactions occur more easily. Under anaerobic conditions, the enzyme activities of reductase and protease are 40–75% higher than under aerobic conditions, and dehydrogenase activity is about 40–60% higher, which is prone to hydrolysis and reduction (Goel et al., 1998). The removal of OPEs under aerobic conditions is greater than that under anaerobic conditions, probably because OPEs can be removed by volatilization under the former, while an anaerobic bottle is a sealed environment in the anaerobic treatment and OPEs, therefore, cannot be removed by volatilization.

### Electron Donors

Addition of electron donors significantly increased the removal of chlorinated OPEs. Reduction dehalogenation is an important anaerobic biodegradation mechanism. Electron donors increase the reduction dehalogenation of chemical substances (Futagami et al., 2010), so the addition of electron donors to sludge may also increase the degradation of chlorinated OPEs. Moreover, organic pollutants can be removed by co-metabolism. For example, adding acetic acid as a co-substrate can increase the bacterial metabolism of triphenyl phosphate (TPP) (Hou et al., 2019), and adding lactic acid can significantly improve the efficiency of anaerobic removal of naphthalene (Cao et al., 2012). As a result, when the intermediate products, including acetate + propionate + butyrate + lactate, are added as a co-matrix, the degradation of chlorinated OPEs is increased by co-metabolism. Studies have also shown that the addition of a common matrix increases the volume of methane production, while methanogenic organisms play an important role in the biodegradation of organic matter and increase the degradation of pollutants (Nazaries et al., 2013).

The removal of triphenyl ester OPEs in anaerobic sludge is relatively fast, so the addition of electron donors does not significantly increase their removal. Microorganisms need time to adapt to electron donors, the adaptation process needs to go through a lag period (Fischer and Majewsky, 2014), and the triphenyl ester OPEs are removed before the end of the lag period.

The addition of electron donors has an inhibitory effect on the removal of alkyl OPEs, which may be due to the fact that the metabolic pathway for removal of alkyl OPEs does not involve co-metabolism, but rather other degradation pathways. Studies of the anaerobic degradation of sediment have also shown that the addition of either acetic acid or lactic acid inhibited the rate of degradation of non-ylphenol (Chang et al., 2004).

### Electron Acceptors

The rates of degradation of triphenyl ester OPEs were slightly accelerated by adding FeCl_3_, but those of other OPEs were not significantly increased. Thus, the mechanism by which FeCl_3_ acts as an electron acceptor for OPEs is unclear. Moreover, FeCl_3_ is also a flocculant, which can reduce the toxicity of wastewater and improve its biodegradability (Karthik et al., 2008). Flocculation can also increase the contact area between sludge and organic pollutants in sewage and accelerate the rate of degradation and increase the percentage removal of triphenyl ester OPEs.

Usually, NaNO_3_ and Na_2_SO_4_ are added as electron receptors to increase pollutant degradation. The presence of NaNO_3_ and Na_2_SO_4_ results in higher bacterial abundance and greater enrichment of functional genes during the nitrogen, carbon, sulfur, and phosphorus cycles (Dou et al., 2008; Xu et al., 2014; Zhang et al., 2019). In river sediments, the presence of nitrate and sulfate was found to accelerate the degradation of polycyclic aromatic hydrocarbons (PAHs) (Yang et al., 2020). However, due to the different reactions of different substances to electron acceptors (Schmidt et al., 2017), we came to different conclusions in our experiments. In this study, NaNO_3_ and Na_2_SO_4_ did not significantly improve the anaerobic removal of triphenyl OPEs, and they inhibited the removal of chlorinated and alkyl OPEs. The concentration of added electron acceptors used in this study has been proven not to inhibit microorganisms (Dou et al., 2008; Guerrero-Barajas et al., 2014; Gou et al., 2019). Therefore, the inhibition of the removal of chlorinated and alkyl OPEs may have been due to the production of toxic intermediate products, which requires further study. Zhang et al. (2011) have also proposed that sulfate can stimulate sulfate-reducing bacteria to compete with methanogenic bacteria for electron utilization to produce sulfides that are toxic to microorganisms (Vogel et al., 1987). Nutrient salts such as magnesium sulfate and ammonium nitrate in the medium also negatively affect the biodegradation of chlorophenol (Sahoo et al., 2010). Nitrate can also reversibly block the metabolism of trimethyltrinitramine (Beller, 2002).

### Surface-Active Agents

When organic matter is hydrophobic, adding surfactant can overcome the diffusion limitation of organic matter to cells, reduce the tension and viscosity of the water interface effectively (Zdarta et al., 2018), increase bioavailability, and thus increase the rate of removal (Varjani and Upasani, 2017). The biosurfactant rhamnolipid and the non-ionic surfactant Tween 80 can both usually increase the contact between microbial cells and the matrix. Previous studies have shown that rhamnolipid can significantly increase the rate of removal of phenanthrene, diesel, and pyrene. The addition of Tween 80 can also significantly increase the biodegradation ratio of phenanthrene (Burgess and Pletschke, 2008; Kang et al., 2019). Rhamnolipid and Tween 80 can also increase fungal biomass, the decomposition of hemicellulose and cellulose, and the rate of decomposition by 8.0 and 11.6%, respectively. They can also significantly affect the rate of carbohydrate and amino acid metabolism (Yin et al., 2019). In addition, they can also increase the maximum speed of enzyme reactions (Mo et al., 2008). However, the effect of surfactants on enzymes is not always positive (Veronika et al., 2018). Zeng et al. (2006) have pointed out that Tween 80 and rhamnolipid can increase the enzyme activity of amylase and xylanase, but have a negative effect on protease.

Addition of rhamnolipid had no significant effect on OPEs removal in this study, except for chlorinated OPEs. Tween 80 has an inhibitory effect on OPE degradation, possibly due to its ability to interact with microbial proteins and manipulate them to alter enzyme conformation, thereby altering enzyme activity, stability, and specificity, as well as its potential toxicity to microorganisms (Van Hamme et al., 2006; Tiehm and Schmidt, 2011).

### Reduction of Fe Powder

Fe powder can significantly increase the rate of removal of OPE triphenyl esters. Zero-valent iron can provide electrons for microorganisms, and it has a high reduction activity that can maintain a low redox potential in the anaerobic environment (Völker et al., 2017) and significantly increase the anaerobic biodegradation of chlorpyrifos to reduce water poisoning (Shi et al., 2019a). Adding zero-valent iron can also increase anaerobic digestion in STPs as well as methane production (Wei et al., 2018). Fe can also significantly alter the bacterial and methanogenic community structure (Pan et al., 2019), is also an important protein cofactor (Kleemann and Meckenstock, 2011), and is essential for most organisms (Beauchene et al., 2015). Feng et al. (2014) have also pointed out that zero-valent iron as a reducing material is expected to enhance anaerobic processes, including hydrolytic acidification, which can help accelerate and improve anaerobic acid production and create good conditions for subsequent treatment (Liu et al., 2012).

### Treatments of Different Aerobic Sludge Concentrations

A comparative study of the removal of OPEs from 1 to 10 g⋅L^–1^ sludge concentrations revealed that the removal percentages of OPEs were not proportional to the sludge concentration. In the early stages of the experiment, triphenyl ester OPEs had significantly higher removal percentages in 1 g⋅L^–1^ than in 10 g⋅L^–1^ sludge concentration, which may have been due to the greater contribution from the removal of triphenyl ester OPEs by volatilization. When the sludge concentration was high (10 g⋅L^–1^), the adsorption of OPEs was greater, which reduced the degree of removal in the early stage. After 16 days, the removal percentages of the three types of OPEs in the higher concentration of sludge (10 g⋅L^–1^) exceeded that of the low concentration (1 g⋅L^–1^), mainly because the higher sludge concentration ensured the diversity of microorganisms made the microorganisms more resistant to toxic pollutants. Currently, the European Union (ECHA, 2017) proposes the use of enhanced biodegradability tests to assess the biodegradability potential of chemicals, that is, by increasing the sludge concentration or increasing the volume of the test solution to ensure the test error caused by the heterogeneity of the dominant species when the sludge is added is reduced as much as possible.

## Conclusion

The biodegradability of nine OPEs in both aerobic and anaerobic activated sludge derived from kitchen garbage biomass and agricultural residues under different conditions was investigated.

Under anaerobic conditions, the removal percentages of triphenyl ester OPEs were significantly higher than those of chlorinated and alkyl OPEs. The addition of electron donors, electron acceptors, surfactants, and Fe powder did not always increase the rate of degradation of the different kinds of OPEs, which is closely related to the structure of the OPEs. The addition of FeCl_3_ and Fe powder increased the rate of degradation of triphenyl ester OPEs, with the DT_50_ of triphenyl ester OPEs being in the range 1.7–3.8 days when FeCl_3_ was added and 1.3–4.7 days when Fe powder was added, compared to a DT_50_ of 4.3–6.9 days for the blank control. Addition of electron donors and rhamnolipid increased the removal of chlorinated OPEs, with the DT_50_ values of TCEP and TDCP being 18.4 and 10.0 days, respectively, after electron donors were added, and 13.7 and 3.0 days, respectively, after rhamnolipid was added. No treatment increased the removal of alkyl OPEs as their low anaerobic degradability prevented that occurring.

The biodegradation rates of OPEs under aerobic conditions were significantly higher than those under anaerobic conditions. The sludge with the higher concentration had a lower rate of degradation for highly adsorbent chemicals at the beginning of the test. However, the sludge with the higher concentration helped the microorganisms present in the sludge to adapt and remove OPEs by the end of test.

These results will provide a new perspective on the effect of activated sludge cultured with kitchen garbage biomass and agricultural residues in eliminating emerging pollutants.

## Data Availability Statement

The original contributions generated for this study are included in the article/supplementary material, further inquiries can be directed to the corresponding author/s.

## Author Contributions

LZ and ZZ proposed the idea. XY and WG did the experiments. JL, LS, and GJ analyzed the results. All authors contributed to the article and approved the submitted version.

## Conflict of Interest

The authors declare that the research was conducted in the absence of any commercial or financial relationships that could be construed as a potential conflict of interest.
